# Coenzyme Q10 levels are low and may be associated with the inflammatory cascade in septic shock

**DOI:** 10.1186/cc10343

**Published:** 2011-08-09

**Authors:** Michael W Donnino, Michael N Cocchi, Justin D Salciccioli, Daniel Kim, Ali B Naini, Catherine Buettner, Praveen Akuthota

**Affiliations:** 1 Department of Emergency Medicine, Beth Israel Deaconess Medical Center, One Deaconess Road, West Clinical Center 2, Boston, MA 02215, USA; 2 Division of Pulmonary, Critical Care and Sleep Medicine, Department of Medicine, Beth Israel Deaconess Medical Center, One Deaconess Road, Boston, MA 02215, USA; 3 Trauma/Surgical/Neurological Critical Care, Department of Anesthesia Critical Care, Beth Israel Deaconess Medical Center, One Deaconess Road, Boston, MA 02215, USA; 4 Department of Pathology and Cell Biology, Columbia University Medical Center, 630 West 168th Street, VC 15-208, New York, NY 10032, USA; 5 Division of General Medicine and Primary Care, Department of Medicine, Beth Israel Deaconess Medical Center, 330 Brookline Ave, Boston, MA 02215, USA; 6 Division of Allergy and Inflammation, Department of Medicine, Beth Israel Deaconess Medical Center, One Deaconess Road, Boston, MA 02215, USA

## Abstract

**Introduction:**

Mitochondrial dysfunction is associated with increased mortality in septic shock. Coenzyme Q10 (CoQ10) is a key cofactor in the mitochondrial respiratory chain, but whether CoQ10 is depleted in septic shock remains unknown. Moreover, statin therapy may decrease CoQ10 levels, but whether this occurs acutely remains unknown. We measured CoQ10 levels in septic shock patients enrolled in a randomized trial of simvastatin versus placebo.

**Methods:**

We conducted a *post hoc *analysis of a prospective, randomized trial of simvastatin versus placebo in patients with septic shock (ClinicalTrials.gov ID: NCT00676897). Adult patients with suspected or confirmed infection and the need for vasopressor support were included in the initial trial. For the current analysis, blood specimens were analyzed for plasma CoQ10 and low-density lipoprotein (LDL) levels. The relationship between CoQ10 levels and inflammatory and vascular endothelial biomarkers was assessed using either the Pearson or Spearman correlation coefficient.

**Results:**

We analyzed 28 samples from 14 patients. CoQ10 levels were low, with a median of 0.49 (interquartile range 0.26 to 0.62) compared to levels in healthy control patients (CoQ10 = 0.95 μmol/L ± 0.29; *P *< 0.0001). Statin therapy had no effect on plasma CoQ10 levels over time (*P *= 0.13). There was a statistically significant relationship between plasma CoQ10 levels and levels of vascular cell adhesion molecule (VCAM) (*r*^2 ^= 0.2; *P *= 0.008), TNF-α (*r*^2 ^= 0.28; *P *= 0.004), IL-8 (*r*^2 ^= 0.21; *P *= 0.015), IL-10 (*r*^2 ^= 0.18; *P *= 0.025), E-selectin (*r*^2 ^= 0.17; *P *= -0.03), IL-1ra (*r*^2 ^= 0.21; *P *= 0.014), IL-6 (*r*^2 ^= 0.17; *P *= 0.029) and IL-2 (*r*^2 ^= 0.23; *P *= 0.009). After adjusting for LDL levels, there was a statistically significant inverse relationship between plasma CoQ10 levels and levels of VCAM (*r*^2 ^= 0.24; *P *= 0.01) (Figure 3) and IL-10 (*r*^2 ^= 0.24; *P *= 0.02).

**Conclusions:**

CoQ10 levels are significantly lower in septic shock patients than in healthy controls. CoQ10 is negatively associated with vascular endothelial markers and inflammatory molecules, though this association diminishes after adjusting for LDL levels.

## Introduction

Severe sepsis and septic shock are major causes of morbidity and mortality. The annual incidence of deaths due to severe sepsis and septic shock is estimated to be over 215,000 in the United States [1]. Organ dysfunction related to sepsis is associated with significant alterations in metabolic pathways and cellular function [2]. Although the exact mechanism by which sepsis leads to organ dysfunction remains unclear, hypoxia and several mediators in the systemic inflammatory response have been shown to directly impair mitochondrial function and may lead to increased morbidity and mortality in patients with septic shock [3-5].

Coenzyme Q (CoQ10), or ubiquinone, is located within the inner mitochondrial membrane and acts as an electron transport mediator from complex I or complex II to complex III [2]. While a small portion of CoQ10 may be obtained from dietary sources (including food and oral supplementation), it is primarily synthesized endogenously in the endoplasmic reticulum from tyrosine and mevalonate and vitamins B_2_, B_9_, B_12 _and C and is transported in the plasma by low-density lipoprotein (LDL) [6]. Low CoQ10 levels may result from impairment in CoQ10 synthesis, decreased dietary intake or increased requirement (such as during conditions that increase oxidative stress), or from any combination of these factors. Previous investigations have demonstrated that prolonged statin use may decrease CoQ10 levels in patients with cardiovascular disease; however, a causal effect of stain therapy on decreased CoQ10 levels remains a topic of debate [7,8]. In addition to the potential negative effect that statins may have on CoQ10 levels, the increased metabolic demand resulting from sepsis may also deplete CoQ10 levels.

Statins may hold therapeutic value for the prevention and/or treatment of patients with sepsis. Previous investigations have demonstrated important immunomodulatory activity of statins on the inflammatory response and direct alterations of leukocyte-endothelial cell interactions [9-11]. Furthermore, statin therapy has been associated with decreased morbidity and mortality in patients with sepsis [12]. However, statins may also decrease levels of CoQ10, an essential component of the respiratory chain, leading to mitochondrial dysfunction [13].

The statin, CoQ10 and sepsis triangulation was recently reviewed, and the lack of human data to support theoretical assumptions and hypotheses was noted. Specifically, Brealey *et al*. [14] stated that low levels of antioxidants cause mitochondrial dysfunction in human sepsis, but there are currently no data to account for the levels of the mitochondrial component CoQ10. Herein we report on the levels of plasma CoQ10 in patients with septic shock and the relationship between CoQ10 and the inflammatory cascade. The primary goal of this investigation was to determine whether patients with septic shock have decreased plasma CoQ10 compared to healthy controls. Our secondary aims were to determine whether CoQ10 levels are associated with the sepsis-induced inflammatory response and vascular endothelial activation and to determine whether acute statin therapy reduces CoQ10 levels [15-19]. We measured CoQ10 levels and inflammatory and vascular endothelial biomarkers in plasma that had been collected in a prospective, randomized trial of statins versus placebo for patients with septic shock.

## Materials and methods

### Study design

We conducted a *post hoc *analysis of a prospective, randomized trial of septic shock patients admitted to an urban university teaching hospital (ClinicalTrials.gov ID: NCT00676897). The initial trial was a prospective, randomized, double-blind, placebo-controlled, single-center trial of the effect of statin therapy for patients with septic shock. Patients enrolled in the trial were given either 40 mg of simvastatin or placebo once daily for a maximum of seven days or until hospital discharge, whichever came first. The primary outcome for the initial trial was the time until shock reversal with a secondary outcome of attenuation of the inflammatory cascade following intervention. The current *post hoc *analysis was performed using clinical and inflammatory biomarker data collected from the patients who were included in the initial trial. The study was approved by the Institutional Review Board at Beth Israel Deaconess Medical Center.

### Study setting and population

All patients who presented to our urban university teaching hospital with suspected or confirmed infection were considered for enrollment. Patients were screened by trained research assistants using the hospital's electronic medical record system in the emergency department and ICUs. Inclusion criteria for the initial trial consisted of adult patients (age > 18 years) with two or more of the four systemic inflammatory response syndrome criteria, suspected or confirmed infection (pneumonia, sepsis, pyelonephritis, and so on) and evidence of shock (defined as the use of vasopressor medication for a minimum of one hour). Exclusion criteria consisted of statin or digoxin use upon presentation, alanine aminotransferase (ALT) and aspartate aminotransferase (AST) levels more than three times the upper limit of normal or creatine phosphokinase level more than four times the upper limit of normal. Patients were also excluded if they did not require vasopressor support. Healthy controls were enrolled if they met the inclusion criteria of age > 18 years and no acute medical or surgical problems that would require hospital admission. Controls were excluded if they had any significant comorbid disease. Written informed consent was obtained from the patients or their designated surrogates prior to any research intervention.

### Interventions

All patients received standard medical therapy for the treatment of the underlying infection as determined by the clinical team. The initial randomized trial included provision of either simvastatin 40 mg or placebo once daily for a maximum of seven days or until hospital discharge, whichever occurred first. Patients included in the *post hoc *analysis were assessed for clinical and biomarker data, including levels of CoQ10 and plasma LDL as well as vascular endothelial and inflammatory markers.

### Data collection

Pertinent patient demographics, including age, gender, race, medical history and suspected source of infection were recorded at baseline. Vital signs and laboratory data, including complete blood count, electrolytes and lactate, were recorded at the time of enrollment and at serial time intervals.

Blood was collected at serial time intervals (0, 24, 48, 72 and 168 hours) into evacuated tubes containing ethylenediaminetetraacetic acid from an existing venous or arterial catheter. The blood was immediately centrifuged at 2,800 × *g *at 4°C for 10 minutes, and 0.5 mL of plasma were transferred into cryogenic vials and stored at -80°C immediately after centrifugation. Samples from patients who had blood collected at both 0 and 72 hours were used in the current biomarker analysis.

### Primary data analysis

#### Cytokine and soluble cardiovascular biomarker measurement

Plasma from patients and controls at 0 and 72 hours was collected and stored at -80°C until thawed to assay for cytokines and soluble cardiovascular biomarkers. Analytes were measured by multiplex analysis (Bio-Plex Laboratories, Hercules, CA, USA) using 96-well multiplex kits (Millipore, Billerica, MA, USA). Analytes measured were IL-1ra, IL-2, IL-6, IL-8, IL-10, TNF-α, vascular endothelial growth factor (VEGF), soluble E-selectin, soluble vascular cell adhesion molecule (VCAM) and soluble intercellular adhesion molecule (ICAM). For vascular biomarkers (E-selectin, VCAM and ICAM), plasma samples were measured at either 1:50 or 1:100 dilution factor. For cytokines and VEGF plasma, samples were measured undiluted. All samples were measured in duplicate (mean values from duplicate results were used for analysis).

#### Plasma coenzyme Q10 measurement

Plasma CoQ10 levels were measured at the Laboratories of Metabolic and Mitochondrial Diseases at Columbia University, where analysis of CoQ10 is performed for both commercial and research purposes. Concentrations of CoQ10 were measured by using high-pressure liquid chromatography (HPLC) with an electrochemical detection system. Briefly, 50 μL of thawed plasma were mixed in an Eppendorf tube with 50 μL of ethanol solution containing 25 ng of CoQ9 (used as internal standard). After the addition of 900 μL of 1-propanol, the tube was vortex-mixed for 2 minutes and then centrifuged at high speed in the cold room for 10 minutes. An aliquot (about 300 μL) of the supernatant was filtered through a 0.2-μm filter column and transferred into an HPLC sample injection vial, and an aliquot of 50 μL of the extract was injected into an HPLC system equipped with a C18 reversed-phase column and an ESA Coulochem II electrochemical detector (ESA, Inc., Chelmsford, MA, USA). The established healthy control CoQ10 concentration range in the literature is 1.04 μmol/L ± 0.33, though we measured a set of healthy controls in this study to provide direct comparison and validate our methods [20].

#### Statistical methods

Basic demographics and patient characteristics are reported using descriptive statistics. Continuous data are reported as medians with interquartile ranges (IQRs), and categorical data are reported as frequency with percentages. As LDL is the primary transport molecule for CoQ10, the ratio of CoQ10 to LDL (CoQ10:LDL) levels was calculated, and these values were used for subsequent analyses. CoQ10 levels in the septic population were compared to healthy controls using the Wilcoxon rank-sum test. The relationship between CoQ10 and inflammatory and vascular endothelial biomarkers was assessed using either the Pearson or Spearman correlation, depending on the normality of the data. Statistical analysis was performed using SAS version 9.2 software (SAS Institute, Cary, NC, USA), and *P *< 0.05 was considered significant for statistical testing of the data.

#### Power calculation

Miles *et al*. [20] previously reported healthy control values for CoQ10 (1.04 μmol/L ± 0.33). Thus, assuming a 30% lower value in septic shock patients with a similar standard deviation, our study has 80% power to detect a difference between groups (α = 0.05) with a sample size of 28 septic patients and 16 controls.

## Results

**Figure 1:**
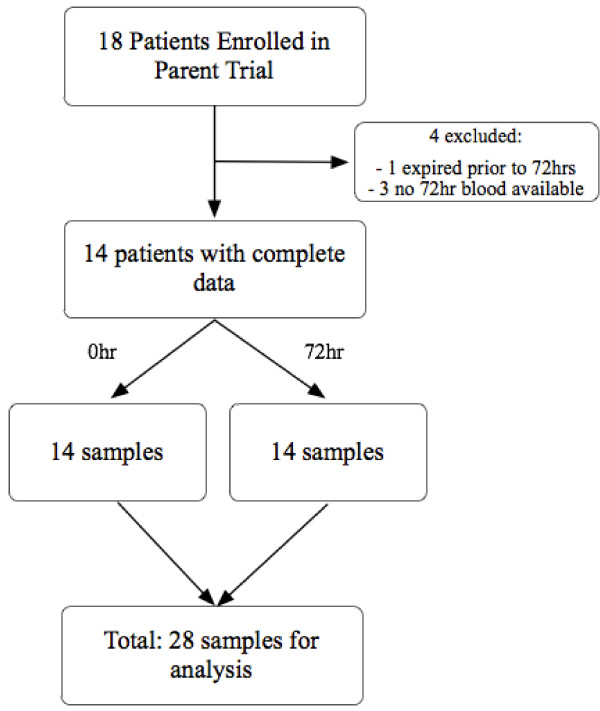
**Schematic of our study design**. A total of 28 samples were analyzed (14 patients with baseline and follow-up blood specimens were included for analysis).

A total of 18 subjects were randomized in the initial trial. Of these 18 patients, one patient died prior to the 72-hour follow-up examination and three had no follow-up blood specimen available and were therefore excluded from the current analysis. Of the 18 patients in the original trial, 14 had CoQ10 levels measured at both 0 and 72 hours, and these 28 samples were included in our analysis (Figure 1). The baseline characteristics of these 14 patients are provided in Table 1. In addition, we measured CoQ10 levels in 16 healthy controls. The median age of the septic shock patients was 55 years (IQR 47 to 62 years), and 64% of them were females. The severity of illness was assessed using the Acute Physiology and Chronic Health Evaluation II (APACHE II) score, with a median score of 19 (IQR 17 to 21). There was no difference in severity of illness between groups in the initial trial on the basis of APACHE II scoring (placebo: 19 (IQR 18 to 20), statin: 17 (IQR 16 to 21)) or lactic acid (placebo: 2.0 mmol/L (IQR 1.9 to 2.2), statin: 2.3 mmol/L (IQR 1.3 to 3.3)).

**Table 1:** Baseline patient characteristics^a^

Demographics	Data (*N *= 14)
Age, years	55 (47 - 62)
Females, *n *(%)	9 (64)
Race, *n *(%)	
African-American	3 (21)
Caucasian	11 (79)
Comorbidities, *n *(%)	
Diabetes	2 (14)
Hypertension	8 (57)
Chronic obstructive pulmonary disease	2 (14)
Coronary artery disease	2 (14)
Congestive heart failure	3 (21)
Renal insufficiency or end-stage renal disease	4 (29)
Initial vital signs	
Temperature (°F)	98.9 (97.0 - 100.0)
MAP (mmHg)	64 (54 - 83)
Heart rate (bpm)	99 (88 - 112)
Respiratory rate (breaths/minute)	20 (16 - 23)
Initial laboratory data	
Sodium (mEq/L)	140 (137 - 140)
Potassium (mEq/L)	3.8 (3.1 - 4.2)
Bicarbonate (mEq/L)	19 (16 - 24)
Creatinine (mg/dL)	1.5 (0.9 - 3.5)
INR	1.2 (1.2 - 1.5)
AST (IU/L)	29 (7 - 35)
ALT (IU/L)	29 (19 - 45)
White blood cells (1 × 10^3^/μL)	10.5 (6 - 16)
Hematocrit (%)	31 (29 - 34)
Platelets (1 × 10^3^/μL)	196 (164 - 302)
Arterial blood gas	
pH	7.36 (7.29 - 7.42)
PaCO_2 _(mmHg)	38 (30 - 51)
PaO_2 _(mmHg)	101 (75 - 113)
Lactate (mmol/L)	2.1 (1.4 - 2.4)
CoQ10 data	
CoQ10 (μmol/L)	0.49 (0.26 - 0.62)
LDL (mg/dL)	42 (35 - 61)
CoQ10:LDL ratio	101 (82 - 150)

^a^ALT: alanine aminotransferase, AST: aspartate aminotransferase, CoQ10: plasma coenzyme Q10, INR: International Normalized Ratio, LDL: low-density lipoprotein, MAP: mean arterial pressure, PaCO_2_, partial pressure of carbon dioxide, PaO_2_: partial pressure of oxygen. Data are medians with interquartile ranges unless otherwise indicated.

**Figure 2:**
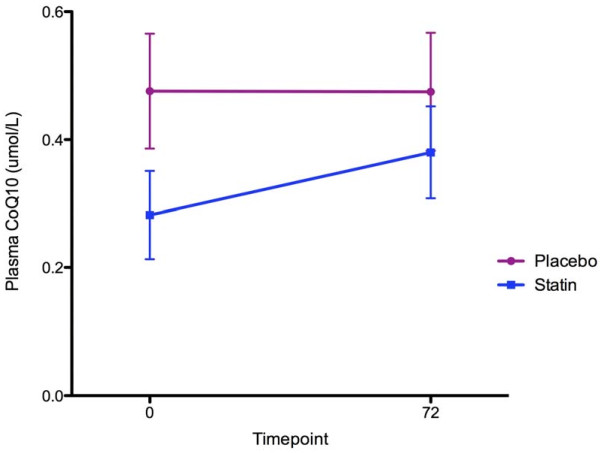
**Comparison of ratios of coenzyme Q10 to low-density lipoprotein (CoQ10:LDL) between randomization groups in the parent trial**. There was no significant difference in the change in plasma CoQ10 between the statin and placebo groups (*P *= 0.13). Bars are Standard Error of the Mean.

**Figure 3:**
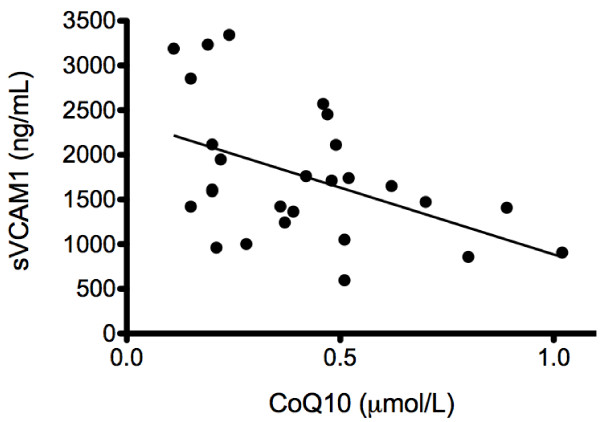
**Association between CoQ10 and sVCAM1, soluble vascular cell adhesion molecule levels (*P *= 0.04)**.

CoQ10 levels for the patients were low, with a median of 0.49 μmol/L (IQR 0.26 to 0.62). The median LDL level in the sample was 42 mg/dL (IQR 35 to 61), and the CoQ10:LDL ratio was 101 (IQR 82 to 150). CoQ10 levels in the patient group were statistically significantly lower than in the 16 healthy controls (0.95 μmol/L ± 0.29; *P *< 0.0001). Of note, our control values of CoQ10 were similar to previously established healthy control ranges, thus validating our methods (1.04 μmol/L ± 0.33 μmol/L) [20]. Randomization from the initial trial had no effect on CoQ10 level (Figure 2). Specifically, there was no significant difference in the change in mean CoQ10 levels between randomization groups (*P *= 0.13), though there appears to have been a slight trend toward decreases in the statin group. There was a statistically significant relationship between CoQ10 level and VCAM (*r*^2 ^= 0.2; *P *= 0.008), TNF-α (*r*^2 ^= 0.28; *P *= 0.004), IL-8 (*r*^2 ^= 0.21; *P *= 0.015), IL-10 (*r*^2 ^= 0.18; *P *= 0.025), E-selectin (*r*^2 ^= 0.17; *P *= -0.03), IL-1ra (*r*^2 ^= 0.21; *P *= 0.014), IL-6 (*r*^2 ^= 0.17; *P *= 0.029) and IL-2 (*r*^2 ^= 0.23; *P *= 0.009). After adjusting for LDL levels, there were statistically significant inverse relationships between CoQ10 and VCAM (*r*^2 ^= 0.24; *P *= 0.01) (Figure 3) and IL-10 (*r*^2 ^= 0.24; *P *= 0.02). Additional biomarkers demonstrated no association with CoQ10 after adjustment for plasma LDL (all *P *> 0.05).

## Discussion

To the best of our knowledge, this is the first report on the levels of CoQ10 in humans with septic shock. In this *post hoc *analysis of a prospective, randomized trial, we found that patients in septic shock have significantly lower levels of plasma CoQ10 compared to a normal healthy control population [20]. Furthermore, we found a statistically significant inverse relationship between plasma CoQ10 levels with inflammatory cytokines and vascular endothelial biomarkers.

CoQ10 is an essential component of the electron transport chain, acting as an electron transporter of mitochondrial complexes I and II to complex III. Disruption of this mechanism can compromise the oxidative capacity of the mitochondria and lead to a decreased level of energy production. Mitochondria are the primary organelles for cellular energy production, and mitochondrial dysfunction can be detrimental in critical illness. The presence of mitochondrial dysfunction in septic shock has been described previously, though the exact components and reasons for this dysfunction are not completely known [3-5]. The hypothesis that CoQ10 deficiency contributes to mitochondrial dysfunction remains plausible. Our findings that plasma CoQ10 levels are abnormally low in a septic shock cohort compared to healthy adults supports this potential link.

Previous research studies have demonstrated that inflammatory cytokines and vascular endothelial markers are abnormally elevated in septic shock, indicating both inflammation and activation of the vascular endothelium. Within septic patients, elevation of these biomarkers has been associated with worse outcomes [15-18]. We found a statistically significant association between CoQ10 levels and VCAM and IL-10; whether the relationship is causal remains unknown. However, CoQ10 treatment in other diseases, such as breast cancer, did show that provision of the compound ultimately resulted in a lowering of biomarkers, thus supporting a causal relationship [21].

The finding of abnormally low CoQ10 levels is consistent with the expected pathophysiology in septic shock. Since LDL is the primary transport molecule for CoQ10 in plasma, we measured LDL levels and found that these levels were low, which is in agreement with reports in the literature on lipoprotein variations in sepsis and septic shock [22]. The decrease in the carrier may lead to depletion of CoQ10. In addition, CoQ10 is partially provided by diet, and patients with critical illness often have nutritional deficiencies for multiple reasons. Furthermore, through a series of complex biochemical reactions, CoQ10 is synthesized endogenously from a number of components, including mevalonate and tyrosine as well as vitamins B_2_, B_9_, B_12 _and C. Patients in septic shock have deficiencies in micronutrients, including B vitamins, and since CoQ10 in the body is primarily derived from endogenous production, metabolic dysfunction leading to decreased production and/or increased utilization is more likely to account for CoQ10 depletion identified in this study [23]. Regardless of etiology, identifying low CoQ10 levels in patients with septic shock is significant, as the compound is essential to mitochondrial function and may play an important role in the pathophysiology of mitochondrial dysfunction in sepsis. In contrast to previous investigations of mitochondrial dysfunction, our finding that CoQ10 is low in sepsis opens the potential for future therapeutic interventions, as CoQ10 can be administered exogenously.

In experimental models, pretreatment with CoQ10 before induction of sepsis restored hepatic ATP levels, suppressed markers of lipid peroxidation and increased survivability [2]. In a canine model of septic shock, Lelli *et al*. [24] found that pretreatment with CoQ10 led to improvements in cardiac output and mean arterial pressure, though they did not find statistically significant differences in several biomarkers, including TNF-α and IL-6. However, they had a very small sample size (10 dogs, 5 in each group), and they indicated that IL-6, for instance, increased by approximately 3,000% over the baseline level (prior to injection with *Escherichia coli*) compared to an approximately 500% increase in the group that was infused with CoQ10 in addition to *E. coli*, even though this did not reach statistical significance with such a small sample size. A relationship might have been found with a larger sample. In addition, there were multiple markers measured in our study that were not measured by Lelli and colleagues [24]. Thus, the improvements in cardiovascular function that these investigators found may be linked to modifications of vascular or inflammatory biomarkers.

We detected no significant effect of statin therapy on plasma CoQ10 levels in our population; however, our sample size was too small to draw any definitive conclusions, and we did note a trend toward decreases in the statin group as compared to placebo. In 1993, Watts *et al*. [25] reported that patients undergoing statin therapy for hyperlipidemia had significantly reduced CoQ10 levels. However, their investigation was cross-sectional in design and thus they could not determine a causal association between statin use and decreased CoQ10 levels.

The current study has several limitations which must be considered when interpreting the data. First, the sample size in our study was small. The comparison of the total data set of 28 patients to 16 healthy controls is likely robust enough to conclude that CoQ10 levels are decreased in sepsis; however the effect of statins on CoQ10 is less clear, particularly because of our small sample size. Second, there were a number of patients in septic shock who were not included in the data analysis, as they did not meet all inclusion criteria for the initial prospective, randomized trial. For example, we defined "shock" as the requirement for vasopressors for a minimum of one hour, and septic patients who were not vasopressor-dependent were not considered for enrollment in the initial trial. Therefore, larger prospective observational trials measuring CoQ10 in this patient population are required.

## Conclusions

CoQ10 levels are abnormally low in patients with septic shock compared to controls. The clinical significance of this abnormality remains unknown but should be explored, given the essential role that CoQ10 has in the electron transport chain. Low levels of CoQ10 may be associated with high levels of inflammatory cytokines and vascular endothelial biomarkers, though adjusting for LDL (carrier of CoQ10) diminishes this association and may explain this finding.

## Key messages

• Plasma CoQ10 levels are low in human septic shock.

• There may be an association between plasma CoQ10 levels and the inflammatory cascade in human septic shock.

• Statin therapy administered to patients in septic shock had no effect on CoQ10 levels.

• Additional research should be conducted to investigate the potential of CoQ10 as a therapeutic agent for patients in septic shock.

## Abbreviations

ALT: alanine aminotransferase; AST: aspartate aminotransferase; APACHE II: Acute Physiology and Chronic Health Evaluation II; CoQ10: coenzyme Q10; ICAM: intercellular adhesion molecule; INR: International Normalized Ratio; IL: interleukin; LDL: low-density lipoprotein; MAP: mean arterial pressure; TNF-α: tumor necrosis factor α; VCAM: vascular cell adhesion molecule; VEGF: vascular endothelial growth factor.

## Competing interests

The authors declare that they have no competing interests.

## Authors' contributions

MWD and MNC designed the study and were responsible for all aspects of this investigation. JS and DK were responsible for primary data collection and substantial portions of the data interpretation and analysis. PA was responsible for vascular endothelial biomarker and inflammatory cytokine assays. AN was responsible for the CoQ10 assays. CB provided oversight on the interpretation of the CoQ10 results. All authors were involved in the writing of the manuscript and approved the final version.
